# Hepatitis B Seroprotection and the Response to a Challenging Dose among Vaccinated Children in Red Sea Governorate

**DOI:** 10.3889/oamjms.2016.043

**Published:** 2016-05-24

**Authors:** Samia M. Sami, Iman I. Salama, Ghada A. Abdel-Latif, Lobna A. El Etreby, Ahmed I. Metwally, Naglaa F. Abd El haliem

**Affiliations:** 1 *National Research Center, Child Health, Cairo, Egypt*; 2 *National Research Center, Community Medicine Research, Cairo, Egypt*; 3 *Faculty of Medicine, Al Azhar University - Microbiology and Immunology, Cairo, Egypt*

**Keywords:** Anamnestic response, Booster dose, Seroprotection, HBV vaccine, Children, Egypt

## Abstract

**AIM:**

To assess the long-term effectiveness of hepatitis B virus vaccine and the need for a booster dose among children who received three doses of vaccine during infancy in Red Sea Governorate.

**METHODS:**

A cross-sectional study was performed. Interviews with children (9 months to 16 years) and parents were done. Blood samples to assess Hepatitis B markers were tested. Children showing no seroprotection received a booster dose to assess their anamnestic response after four weeks and one year later.

**RESULTS:**

None of the participants had evidence of chronic Hepatitis B. The seroprotection rate was 23.3% and it significantly decreased with age. Multivariate logistic analysis revealed that older age was the significant predicting variable for having no seroprotective level, while baseline anti-HBs level < 3.3 IU/L was the predicting variable for not developing early anamnestic response or loss of late anamnestic response.

**CONCLUSION:**

Long-term immunity persists among children who received complete series of hepatitis B vaccination during infancy even in absence or reduction of anti-HBs over time. Therefore, a booster dose is not necessary to maintain immunity till the age of sixteen.

## Introduction

HBV infection is a leading cause of acute and chronic liver disease, cirrhosis and hepatocellular carcinoma worldwide. WHO estimates that globally, about 2 billion people have been infected with hepatitis B virus, more than 350 million are chronically infected, and nearly 1 million die every year from acute or chronic sequelae of primary infection with the disease [1, 2]. In Egypt, 75%–85% of patients with the chronic liver disease have HBV or HCV infection as a contributing cause [3].

Mass vaccination of neonates and pre-school children has been strongly recommended by the WHO [4, 5]. Hepatitis B vaccines are highly effective and safe and the public should be educated about the importance and necessity of hepatitis B prevention by vaccination [6, 7].

It has been demonstrated that 90–99% of healthy neonates, children, adolescents, and adults develop protective levels of anti-HBs following a standard vaccination course with hepatitis B vaccine. Serum levels of anti-HBs ≥ 10 IU/L are considered to be protective against HBV infection [7-9]. Loss of detectable concentrations of antibodies (anti-HBs) to hepatitis B surface antigen (HBsAg) does not necessarily indicate loss of immunity. The presence of HBV-specific immune memory can be demonstrated by administering a challenging (booster) dose of vaccine and measuring anti-HBs response. A rapid increase in anti-HBs represents an anamnestic response and is considered to indicate the presence of HBV-specific immune memory [10].

In Egypt, HBV vaccination was included in 1992 in the Expanded Program on Immunization (EPI) with injections at 2, 4 and 6 months of age using the recombinant vaccine. The Ministry of Health and Population conducted a wide range of prophylactic strategies to control viral hepatitis. It was reported that the prevalence of hepatitis B surface antigen (HBsAg) positivity among healthy individuals decreased from 10.1% in 1985 to 1.18% in 2008, and the frequency of acute HBV infection as a cause of symptomatic hepatitis decreased significantly from 43.4% in 1983 to 28.5% in 2002 [11, 12].

The aim of the present study is to assess the long-term effectiveness of HBV vaccine and the need for a booster dose among fully vaccinated children during infancy in Red Sea Governorate.

## Subjects and Methods

The present study is a part of a national community based multi-stage cluster sampling design carried out in the period from July 2010 to June 2013 in 6 governorates representing all geographic regions of Egypt. For sampling process and cluster selection, probability proportional to size sampling was used. The sample frame for the current survey was based on the most recent population census 2006. According to the population size of each governorate, the number of participating clusters was identified in each governorate. First, implicit stratification by geographic location in each governorate, lists of shakhas, medinas, and villages were arranged in serpentine order geographically. This stratification was done independently for urban and rural areas. A sampling interval was calculated and accordingly, a random number was selected using a table of random numbers. From these lists, areas such as villages or city blocks were selected. In each selected area, lists of child health center (MCH), kindergarten and school facilities were identified and 5 facility clusters were randomly selected.

The current work presented the part of the project results concerning Red Sea Governorate. From this governorate, 189 children from Hurghada were recruited. The study was conducted at facility levels, where 5 facilities (one maternal and child health center (MCH) or health unit, one kindergarten and 3 schools (1 primary, 1 preparatory and 1 secondary school) were randomly selected from each area according to the age of the targeted children.

The study protocol was approved by the ethical committees of Ministry of Health, National Research Center and Ministry of Education. A close-ended questionnaire was designed and tested. For quality assurance, training sessions for supervisors, interviewers and Ministry of Health staff in Red Sea governorate were carried out. Peel-off barcode sheets were prepared and used for easy tracking of blood samples and laboratory results. Inclusion criteria were children aged from 9 months to 16 years and had received the full 3 compulsory doses of HBV vaccine during infancy. Signed written consents were obtained from each guardian Face to face interview was carried out with the parents or caretakers of the children. Adolescents above 10 years were also interviewed after their verbal assents which were obtained in the presence of their parents or class teachers. The questionnaire was used to collect data about child age, sex, date of birth and other demographic and socioeconomic variables. Data were also collected concerning children’s’ HBV vaccination and the available vaccination cards were revised for date and dose intervals of HBV vaccine. Socioeconomic status (SES) was determined according to Fahmy and El Sherbiny [13]. It depends on parents’ education, maternal working status, water source, sewage disposable, and availability of electricity and some modification of family income. Non-responders for the booster dose were further given 2 additional doses of HBV vaccine with 2 months interval in between.

Out of 88 children proved to have non-seroprotective levels, 45 children participated and accepted to receive a booster dose of 10 μg monovalent Euvax HB vaccine intramuscularly in the front of the left thigh for children < 3 years and in the deltoid muscle for older children. A blood sample was withdrawn from each child for quantitative detection of anti-HBs one month and one year post–booster in order to assess their early and late anamnestic responses respectively. An anamnestic response was defined as rising in anti-HBs to ≥10 IU/L [14]. Three children who did not develop post-booster anti-HBs level ≥ 10 IU/L were then offered two additional doses of Euvax HB vaccine (2nd vaccination series) 1 and 6 months post booster. One month later, a blood sample was withdrawn from only 2 children for quantitative detection of anti-HBs to assess their immune response.

### 

#### Blood sampling and Laboratory Analysis

About 3-5 ml blood sample was withdrawn aseptically from each participant. Serum samples were aliquoted into two labeled sterile cryo tubes and were stored at –20°C until laboratory examination. HBV markers detection was carried out in the Virology lab in the Microbiology Department, Faculty of Medicine (for Girls), Al-Azhar University, Cairo, Egypt. It was carried out using commercially available enzyme-linked immunoassays (ELISA, DiaSorin-Italy) and according to the manufacturer instructions. Quantitative detection of serum anti-HBs and qualitative determination of serum Total HBV core antibody (anti-HBc) and hepatitis B surface antigen (HBsAg) was assessed. According to international standards, anti-HBs ≥ 10 IU/L was considered to be protective against HBV infection [14, 15]. Vaccines that developed an anti HBs level between 10-100 IU/L after the full vaccination dose are referred to as low responders while those above 100 IU/L are good responders [16]. Sero-positivity is defined as cut-off anti-HBs of 3.3 IU/L [17]. Breakthrough infection was defined as anti-HBs seropositivity in vaccinated subjects who were not chronically infected [18].

#### Data analysis and presentation

Data entry was carried out on excel sheet and statistical analysis was performed using SPSS software program version 18.0 (SPSS Inc., Chicago). The geometric mean titer (GMT) was calculated to indicate the central tendency of anti-HBs titers in consideration of the skewed distribution of anti-HBs level. For the calculation of the GMT, children who had an undetectable anti-HBs titer were assigned a nominal serum anti-HBs titer value of 0.05 IU/l [13]. The w2-test was performed for qualitative data and was presented by numbers and percentages. The t-test was performed for comparison between two means and one-way analysis of variance for more than two means. When data were not normally distributed, Mann-Whitney U test was used. The multivariate logistic analysis was performed to predict the risk factors significantly associated with non seroprotection and non-development of early and late anamnestic response. P value was considered statistically significant when its value was less than 0.05 and considered statistically highly significant when its value was less than 0.01.

## Results

The total number of studied children was 189 (100 boys and 89 girls) aged 9 months to 16 years with a mean age of 9.6 years. None of the participants had an evidence of chronic HBV or breakthrough infection. Table 1 shows that the non-seroprotection level significantly increased with age (it was 17.2% for the age group < 5 years and 66% for the age group >10 years, P < 0.05). There was no significant difference in the seroprotection level as regards gender, socioeconomic status, and other studied variables.

**Table 1:** Sero-protection rate among the studied children

Risk Factor	Total n= 189	Baseline anti-HBs level (IU/L)		

		Non sero-protection Anti-HBs <10 n= 88 n (%)	Sero-protection Anti-HBs ≥10 n= 101 n (%)	P value
Gender				
Boys	100	46 (46.0)	54 (54.0)	0.87
Girls	89	42 (47.2)	47 (52.8)	

Age group				
<5years	58	10 (17.2)	48 (82.8)	
5-10years	37	16 (43.2)	21 (56.8)	< 0.001
>10years	94	62 (66.0)	32 (34.0)	

Socioeconomic status				
Very low	28	17 (60.7)	11 (39.3)	
Low	37	18 (48.6)	19 (51.4)	
Middle	47	22 (46.8)	25 (53.2)	0.317
High	70	28 (40.0)	42 (60.0)	

HAZ				
> -2	159	76 (47.8)	83 (52.2)	0.736
≤ -2	22	8 (36.4)	14 (63.6)	

WAZ				
> -2	176	81 (46.0)	95 (54.0)	0.189
≤ -2	3	1 (33.3)	2 (66.7)	

Lactation				
Breast fed	134	57 (42.5)	77 (57.5)	0.272
Artificially fed	29	17 (58.6)	12 (41.4)	

History of hospital admission				
Yes	58	33 (56.9)	25 (43.1)	0.058
No	131	55 (42.0)	76 (58.0)	

History of abscess incision				
Yes	12	5 (41.7)	7 (58.3)	0.714
No	174	82 (47.1)	92 (52.9)	

HAZ = height for age Z score; WAZ = weight for age Z score.

**Figure 1:**
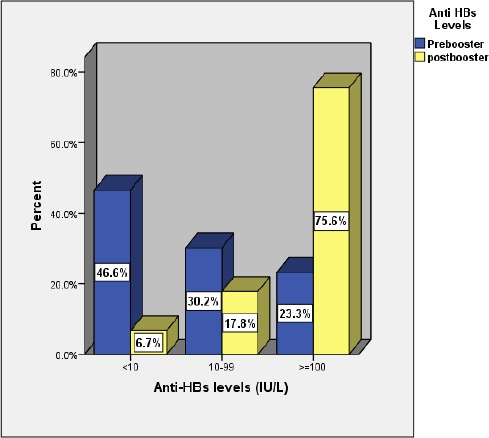
*Baseline seroprotection and early anamnestic response rates among the studied children*.

Only 45 children with non-seroprotection level received the booster dose and 93.4% of them developed an anamnestic response. Figure 1 presents the baseline seroprotection rate and post-booster anamnestic response rate among the studied children. At baseline, out of 189 studied children, only 44 (23.3%) had a good level of seroprotection (anti-HBs ≥ 100 IU/L) and 88 (46.6%) had a non-seroprotective level (< 10IU/L). As regards early post-booster anamnestic response, 75.6% developed a good anamnestic response, 17.8% developed low anamnestic response (10-99 IU/L) and 6.7% were non-responders.

Out of 42 children who developed early, anamnestic response 41 (97.6%) were followed up one year later to assess the persistence of response (late anamnestic response). It was found that 31 children (75.6%) still retained their anamnestic response (anti-HBs ≥10IU/L) while 10 children (24.4%) had lost their protective level.

Meanwhile, the two children who received the 2nd vaccination series developed a good level of immune response (anti-HBs ≥100 IU/L). Both were boys aged 11 years.

Table 2 presents baseline, early and late anamnestic response anti-HBs GMT in relation to gender, age, and pre-booster level. There was no difference between both sexes as regard pre-booster and post- booster (early and late anamnestic response) GMT anti-HBs. While children aged ≤ 10 years had significantly higher pre-booster anti-HBs GMT than older children, post-booster (early and late) GMT anti- HBs did not differ among both age groups. When pre-booster non-seroprotection anti-HBs levels were divided into seronegative (< 3.3 IU/L) and seropositive (≥ 3.3 IU/L), the post-booster early anamnestic response GMT was significantly higher among children with seropositive level of anti-HBs than among those with seronegative level of anti-HBs (848.6 ± 1.3 & 64.3 ± 18.0 respectively) P = 0.004. However, there was no statistically significant difference between both groups for late anamnestic response GMT.

**Table 2:** Anamnestic response and anti-HBs GMT in relation to gender, age, and pre-booster level

Risk Factor	Baseline anti-HBs	Early Anamnestic Response	Late Anamnestic Response

	N = 189 GMT ± SD	N = 45 GMT ± SD	N = 41 GMT ± SD
Gender			
Boys	9.8 ± 13.5	209.6 ± 12.2	95.5 ± 21.3
Girls	12.1 ± 14.9	249.0 ± 9.2	39.3 ± 2.3
*P value*	*0.589*	*0.624*	*0.117*

Age group			
≤10 years	45.7 ± 9.5	227.0 ± 3.9	38.8 ± 12.6
>10 years	3.6 ± 10.9	228.2 ± 12.4	67.6 ± 25.5
*P value*	*<0.001*	*0.189*	*0.409*

Pre-booster level			
< 3.3 IU/L	1.2 ± 1.1	848.6 ± 1.3	10.9 ± 35.7
≥ 3.3 IU/L	6.0 ± 1.4	64.3 ± 18.0	37.5 ± 31.2
*P value*	*0.001*	*0.004*	0.375

GMT=geometric mean titre.

Multivariate logistic analysis revealed that older age was the only significant predicting variable for having no seroprotective level, with AOR = 3.6 and 8.5 among children aged 5-10 and older age respectively compared to those < 5 years, P < 0.01. The significant predicting variable for not developing an early anamnestic response or loss of late anamnestic response was baseline anti-HBs level < 3.3 IU/L with AOR 4.7 & 2.0 respectively and P<0.001 for both (Table 3).

**Table 3:** Predictors of not seroprotection, early and late anamnestic responses using logistic analysis

Variables	AOR	95% CI		P value

		Lower	Upper	
At Baseline				
Age group				
< 5 years	®			
5-10 years	3.616	1.363	9.592	0.01
>10 years	8.509	3.648	19.848	< 0.001

Early Anamnestic Response				
Baseline anti-HBs level				
≥ 3.3 IU/L	®	2.1	10.4	< 0.001
< 3.3 IU/L	4.7			

Late Anamnestic response				
Baseline anti-HBs level				
≥ 3.3 IU/L	®			
< 3.3 IU/L	2	1.4	3	< 0.001

® reference group; AOR: adjusted odds ratio; CI: confidence interval.

## Discussion

Universal infant vaccination will be the key to the elimination and subsequent eradication of hepatitis B. Elimination and eradication of hepatitis B require long-term commitment all over the world to continue the vaccination. Further progress towards the elimination of HBV transmission will require sustainable vaccination programs with improved vaccination coverage [6, 19].

In the current study, 189 studied children received three doses of HB vaccine during infancy and none of them had an evidence of chronic HBV or breakthrough infection. The overall seroprotection rate among all studied children was 53.4%. This rate is similar to that of a study carried out in Dakahlya Egypt where seroprotection rate was 57.7% [20] and in Turkey [21]. However, higher percentages were reported among Chinese, Italian and Iranian children (66.4%,64% & 81% respectively) [9, 22, 23]. The present study also found that 82.8% of participants aged less than 5 years were HBV seroprotected and the seroprotection rate significantly decreased with age which is in agreement with Yazdanpanah et al, [24]. Lower percentages (68.1%) were reported by Zhu et al for the same age group [9]. For older children ≥ 6 years, Liop et al and Behjati et al reported seroprotection rates were >70% [25, 26].

The seroprotection rate was higher among boys (54.0%) than girls (52.8%) and among children with higher socioeconomic status (60.0%) compared with others with very low socioeconomic status (39.4%), but with no statistically significant difference. Similarly, no statistically significant differences were reported as regards gender [5, 21, 22, 24]. However, Sami et al found that in Gharbeya Governorate in Lower Egypt among 762 children, the non seroprotection rate was significantly higher among girls (50.1%) compared to boys (33.6%) (P<0.0005), while the seroprotection rate was found to be significantly higher among high socioeconomic level individuals (61.2%) compared to those of very low class (49.6%) (P < 0.05) [27].

Another Egyptian study carried out on 64 children found that the nonsero-protective rate of anti-HBs was significantly higher among girls than boys aged > 6 years, while no statistical difference was found among children < 6 years [28].

The present study showed that 46.6% of studied children had non-seroprotective levels of anti-HBsat baseline and 93.4% of those receiving the booster dose developed an anamnestic response. Seventy-five percent of them developed good anamnestic response (anti-HBs ≥ 100 IU/L) and 6.7% were non-responders. Similarly, in Egypt, it was found that 91.7% of non-seroprotected children developed anamnestic response and 77.8% of them reported good response [27]. Moreover, Eldesoky et al found that 97.2% of the non-seroprotected children developed good anamnestic response [7]. A Turkish and an Italian study also reported that over 95% of non-seroprotected children developed an anamnestic response post booster [21, 22]. A lower percentage of children developed a post-booster anamnestic response in Iran (78.1%) and in Taiwan [24, 29]. Booster doses of hepatitis B vaccine after primary vaccination in immune competent individuals are currently not recommended by either the WHO or the European Consensus group on Hepatitis B Immunity. The decrease of antibody concentrations below seroprotection level or even below detection levels is not considered as an indicator of loss of protection [30]. The real threat may emerge when the vaccinated subjects begin to engage in high-risk behaviors for HBV transmission in areas of high endemicity. Boosters for certain high-risk groups or for individuals living in the areas of high endemicity may be a reasonable alternative [31-33].

In this study, post booster anamnestic was detected among 100% of non-seroprotected children having pre-booster level ≥ 3.3 IU/l compared to only 90.3% of those having pre-booster level < 3.3 IU/l (P > 0.05). Similarly but to a less extent, Steiner et al, reported that 90.5% of children who had anti-HBs antibody concentrations > 3.3 IU/l reached the seroprotection level after receiving the booster dose compared to 81.0% of seronegative children [34].

In the current study, girls had higher pre-booster and post-booster anti-HBs GMT compared with boys but with no statistically significant difference between both sexes (P > 0.05]. While children aged ≤ 10 years had significantly higher pre-booster anti-HBs GMT than those aged >10 years (P < 0.05], but there was no difference between both age groups in the post-booster anti-HBs GMT. These results are similar to those of other studies in Alaska, Turkey, and Iran which found that pre-booster anti-HBs GMT significantly decreased with age [10, 21, 24]. However, in two Iranian studies, it was found that the post-booster anti-HBsGMT among girls was significantly higher than among boys (P < 0.05] [23, 24]. The statistically significant difference between the mean antibody concentration before and after the booster dose shows the vigorous response of the immune system to the booster and suggests that the immunologic memory is good [24].

In the current study, 100% of the children ≤ 10 years and 91.1% of adolescents developed anamnestic response (P > 0.05). Similarly, in Alaska, 99% of children and 83% of adolescents [10] and 100% of children in Taiwan [32] developed an anamnestic response to a booster dose. Similar results were also reported by [21, 24]. They also found that there was no significant difference in post-booster anamnestic response rate as regards age. In contrast, the anamnestic response rate in Alaskan children decreased according to increasing age [10].

In the current study, the anamnestic response rate to the booster dose did not vary between boys and girls (91.3% and 95.5% respectively, P > 0.05). Similarly, in Gharbeya governorate, the anamnestic response rate was 90.1% among boys and 92.5% among girls, P > 0.05 [27]. On the contrary, Su et al found that Taiwanese adolescent females always had a greater anamnestic response rate than males (P < 0.001) [35].

In the present study, 75.6% of children still retained their anamnestic response after one year post- booster. This was also found in a study in Gambia where 80% of boosted participants still had detectable antibodies [8] and in Canada where 99.3% of subjects in the Engerix-B 10 [mu]g (EB) group and 100% in the Recombivax-HB 2.5 [mu]g (RB) group had detectable anti-HBs one year after the booster [31].

In this study after the booster dose was administered, 6.7% of children were non-responders and two out of the three children who failed to generate an early anamnestic response after receiving the booster dose, were offered another two doses of recombinant HBV vaccine. While 3% of Italian children and 4% of Italian recruits remained negative for anti-HBs, they offered to complete the second course of vaccination with two additional vaccine doses given at 1 and 6 months after the first booster injection [22]. Also, 3% of boosted participants in Gambia did not mount a detectable antibody response following a booster dose of HBV [8]. Samandari et al and Su et al reported that 28.7% and 31% of Alaskan and Taiwanese adolescents respectively did not respond to a booster dose. [10, 35]

Logistic analysis for determining predictors of not seroprotection revealed that older age was the only significant predicting variable for having no seroprotective level while significant predicting variable for not developing an early anamnestic response or loss of late anamnestic response was baseline anti-HBs level < 3.3 IU/L. On the contrary, there was no significant difference as regards HBV booster non-response in subjects below or above 16 years of age in a study by Lu et al [32]. In another study by Wang and Lin, risk of non-response to booster vaccination was highest among adolescents who smoked cigarettes and chewed betel- quid [36].

The study concluded that long-term immunity exists among children who had complete series of hepatitis B vaccination during infancy even in the case of reduced or absent anti-HBs over time. HBV vaccine is highly protective against HBV infection as evidenced by the absence of HBV infection in the vaccinated groups. The longer the time lapse after vaccination, the lower the seroprotection rate and the lower the mean anti-HBs. More than 93% of vaccinated individuals who lost protective levels of antibody developed a rapid anamnestic response when boosted and this indicates the presence of immune memory. Therefore, using routine booster doses of hepatitis B vaccine is not necessary to sustain at least 16 years of protection in immunized individuals.
